# Causal Effect of Pulmonary Hypertension on Brain Structure: A Mendelian Randomization Study Combined With Brain Magnetic Resonance Imaging

**DOI:** 10.7759/cureus.95493

**Published:** 2025-10-27

**Authors:** Anqi Liu, Hongyi Wang, Linfeng Xi, Jie Du, Jianping Wang, Yifei Ni, Shuai Zhang, Peiyao Zhang, Min Liu

**Affiliations:** 1 Radiology, China-Japan Friendship Hospital, Chinese Academy of Medical Sciences and Peking Union Medical College, Beijing, CHN; 2 Pulmonary and Critical Care Medicine, Center of Respiratory Medicine, China-Japan Friendship Hospital, Chinese Academy of Medical Sciences and Peking Union Medical College, Beijing, CHN; 3 Pulmonary and Critical Care Medicine, Center of Respiratory Medicine, State Key Laboratory of Respiratory Health and Multimorbidity, China-Japan Friendship Hospital, National Center for Respiratory Medicine, Beijing, CHN; 4 Radiology, China-Japan Friendship Hospital (Institute of Clinical Medical Sciences) Chinese Academy of Medical Sciences and Peking Union Medical College, Beijing, CHN; 5 Radiodiagnosis, Capital Medical University, Beijing, CHN; 6 Radiodiagnosis, China-Japan Friendship Hospital (Institute of Clinical Medical Sciences) Chinese Academy of Medical Sciences and Peking Union Medical College, Beijing, CHN; 7 Pulmonary Embolism and Pulmonary Hypertension, China-Japan Friendship Hospital (Institute of Clinical Medical Sciences) Chinese Academy of Medical Sciences and Peking Union Medical College, Beijing, CHN; 8 Pulmonary Embolism and Pulmonary Hypertension, National Center for Respiratory Medicine, State Key Laboratory of Respiratory Health and Multimorbidity, National Clinical Research Center for Respiratory Diseases, Beijing, CHN; 9 Radiodiagnosis, State Key Laboratory of Respiratory Health and Multimorbidity, China-Japan Friendship Hospital, Beijing, CHN; 10 Radiology, State Key Laboratory of Respiratory Health and Multimorbidity, China-Japan Friendship Hospital, Beijing, CHN

**Keywords:** brain, chronic thromboembolic pulmonary hypertension, magnetic resonance imaging, mendelian randomization, pulmonary arterial hypertension

## Abstract

Background: The causal effect of pulmonary hypertension (PH) on brain structures remains unknown. Our objective is to study the causal association between pulmonary arterial hypertension (PAH), chronic thromboembolic pulmonary hypertension (CTEPH), and brain structures using a Mendelian randomization approach combined with brain magnetic resonance imaging (MRI) and to analyze the mechanism by which PH affects the brain.

Methods: We analyzed genome-wide association study results from 1,970 patients and 10,363 controls and from 277 patients and 316,345 controls for genetically predicted CTEPH and PAH, respectively. Brain MRI data from the ENIGMA consortium were included as outcomes. Inverse-variance weighting was used to estimate causal associations among CTEPH, PAH, and brain changes, accounting for heterogeneity and pleiotropy. Furthermore, network mediation estimations were performed to analyze the possible mechanisms underlying the impacts of CTEPH and PAH on brain changes.

Results: No heterogeneity or horizontal pleiotropy existed in this Mendelian randomization analysis. CTEPH changed the entorhinal surface (β: 1.13 mm^2^, *p*: 0.047) and inferior parietal surface (β: 10.98 mm^2^, *p*: 0.022) on brain MRI. CTEPH could accelerate the increase of lateral ventricular volume (β: 22.56 mm^3^/year, *p*: 0.039). For PAH, the year-change rate of cerebral white matter volume can be altered (β: -52.72 mm^3^/year, *p*: 0.033). Different circulating immune cells mediated the distinct effects of CTEPH and PAH on brain structure.

Conclusions: CTEPH and PAH exhibit causal associations with distinct brain regions and electrical activity, indicating a varied lung-brain axis linked to the different subtypes of PH and seemingly associated with disturbances in circulating immune cells.

## Introduction

Chronic thromboembolic pulmonary hypertension (CTEPH) and pulmonary arterial hypertension (PAH) are different subtypes of pulmonary hypertension (PH) and have been reported as risk factors for neuropsychiatric disorders [1-4]. Neuropsychiatric disorders are associated with low life quality and high mortality in patients with CTEPH or PAH [5,6]. Although both CTEPH and PAH lead to elevated pulmonary artery pressure, right heart failure, and death [7], CTEPH is caused by pulmonary thromboembolism (PTE), while PAH is characterized by pulmonary arterial remodeling.

Furthermore, patients with CTEPH and PAH have distinct demographic characteristics [8,9] and different frequencies of neuropsychiatric disorders [2,6,10], indicating that these two subtypes of PH may have distinct patterns and mechanisms that affect the lung-brain axis. Early evidence that cortical thickness and surface area of the brain are altered in patients with PAH [11]. However, whether CTEPH and PAH are causally linked to distinct patterns of brain structure or function remains unexplored.

Moreover, studies have shown that CTEPH and PAH patients have elevated levels of inflammatory cytokines [12]. But whether circulating immune cells explain the different impacts on the brain needs to be uncovered. Recently, a large-scale genome-wide association study (GWAS) revealed CTEPH is a partially heritable polygenic disease [13]. This genetic evidence may be used to explain the common or unique effects of CTEPH and PAH on the brain. Mendelian randomization [14-17] employs genetic tools as proxies for exposure to risk factors associated with diseases. These exposures are randomly assigned at the time of pregnancy, serving as a representation of exposure, thereby overcoming the inherent confounding biases present in observational studies.

Thus, we design a two-sample Mendelian randomization study using brain MRI data to document patterns of CTEPH and PAH in brain structure. Furthermore, to analyze the intrinsic mechanisms affecting the lung-brain axis associated with CTEPH and PAH, we search for circulating immune cells as potential mediators of the relationship between CTEPH or PAH and brain structures.

## Materials and methods

Study design

**Figure 1:**
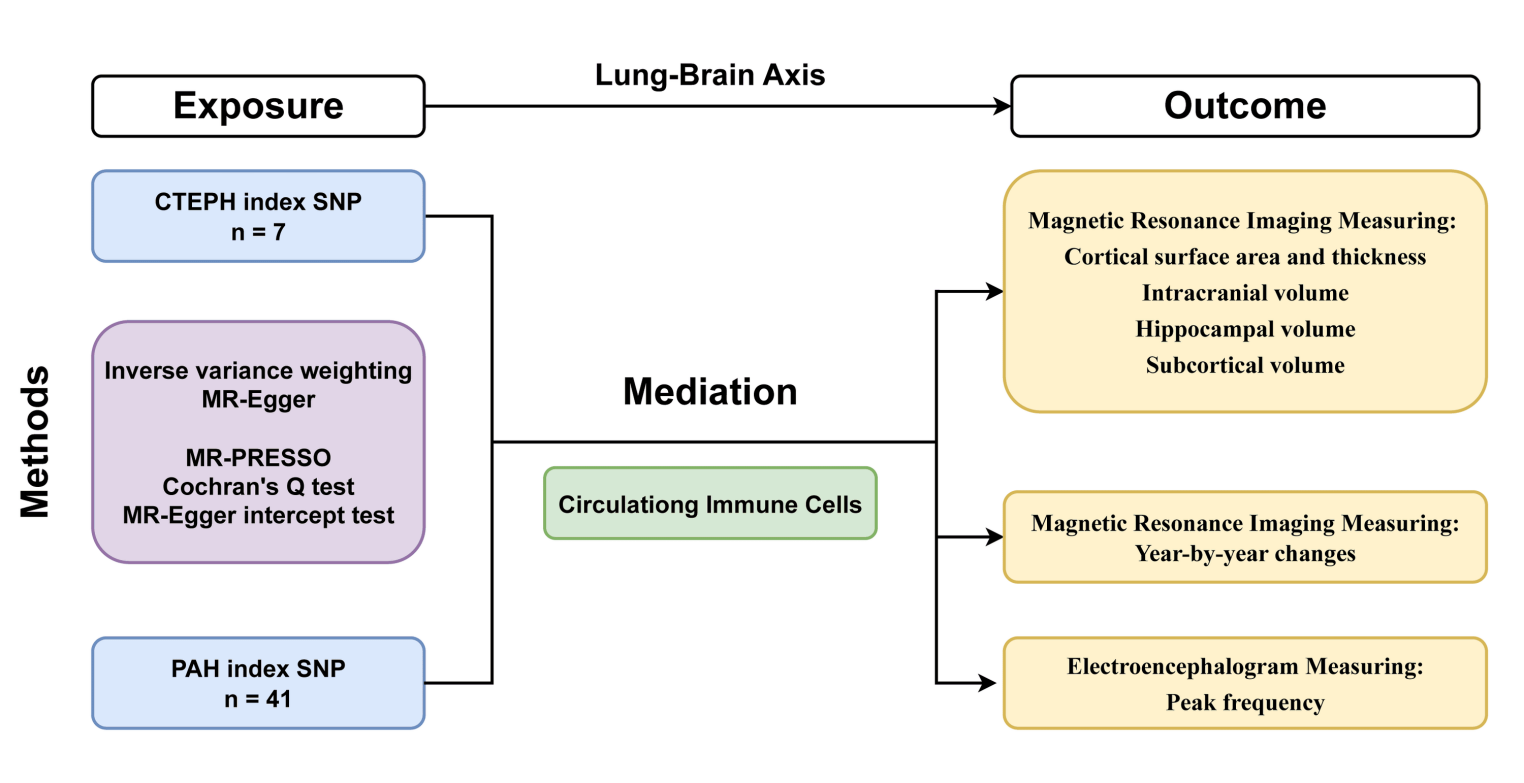
Flowchart of the Mendelian randomization study. Study flowchart of the Mendelian randomization study revealing the causal relationship between PAH, CTEPH, and the brain cortical surface area, thickness, intracranial volume, hippocampal volume, subcortical volume, longitudinal changes (year-by-year changes) with aging, defined using brain MRI, and the electric activity of the brain measured by peak frequency of electroencephalogram. PAH: pulmonary arterial hypertension; CTEPH: chronic thromboembolic pulmonary hypertension; MR: Mendelian randomization; SNP: single-nucleotide polymorphism; MR-PRESSO: Mendelian randomization pleiotropy residual sum and outlier. Flowchart created by the authors.

The present study used GWAS results derived from published studies, and approval was obtained from the appropriate ethics committees for the conduct of these studies; therefore, separate ethical approval was not required for this study. Figure 1 shows the study flowchart of the lung-brain axis between CTEPH, PAH, and brain structures on brain MRI.

The data source for CTEPH and PAH

CTEPH data were obtained from the latest Durham University study, which included 1,970 CTEPH cases and 10,363 controls from a European population with 4,655,481 single-nucleotide polymorphisms (SNPs) [13]. Moreover, we collected summary-level GWAS data on PAH comprising 277 cases and 316,345 controls from the most recent FinnGen data release (June 24, 2024, 11th Release) [18].

Brain structure data on MRI

The cortical surface area and thickness, intracranial volume, hippocampal volume, and subcortical volume in this study are the result of GWAS after quantification of whole-brain MRI by the ENIGMA consortium (https://enigma.ini.usc.edu/research/download-enigma-gwas-results/). They included 51,665 individuals across 60 cohorts and used FreeSurfer MRI processing software to quantitatively analyze T1-weighted (T1WI) sequences. The reported anatomical structures of the gyri were used to determine cortical boundaries, and the Desikan-Killiany map was used to divide the 34 regions of the cerebral cortex. This study used only European-ancestry meta-analysis results, which included two groups: globally corrected and uncorrected. Globally corrected cortical surface area and thickness data exclude the effects of whole-brain surface area or thickness on the surface area or thickness of specific regions. Moreover, GWAS results on the peak frequency of the electroencephalogram and year-by-year changes in brain structure were obtained from the ENIGMA consortium.

Selection of genetic instrument variables

CTEPH and PAH were receptively used as the main exposure, whereas brain structure on brain MRI was used as the outcome. First, we selected SNPs associated with CTEPH and PAH using a significance p-value less than 5×10⁻⁵. Next, we excluded a subset of SNPs based on exposure sample and outcome sample allele identity, palindromic allele, or linkage disequilibrium. The calculated threshold for linkage disequilibrium was 0.001, and linkage disequilibrium was verified within 1 MB upstream and downstream of each SNP. Then, we used LDlink (https://ldlink.nci.nih.gov/?tab=ldtrait) to ensure that no SNP was associated with potential confounding factors, including age, sex, smoking, obesity, and psychiatric disorders. Finally, we calculate the F-value to assess the strength of the selected SNPs as instrumental variables.

Mendelian randomization analyses

Random-effects inverse-variance weighting (IVW) was used as the primary outcome to assess the potential causal relationship among CTEPH, PAH, and cortical structural abnormalities. IVW is a common method used in MR studies to estimate the causal effect by combining the results from multiple genetic instruments. It assumes that the genetic instruments are valid (i.e., they are associated with the exposure, independent of confounders, and affect the outcome only through the exposure). The IVW method provides a weighted average of the causal estimates from each genetic instrument. MR-Egger and weighted median methods were used to further validate the results of IVW in case the instrumental variables had undetected potential pleiotropy or there were potentially invalid instrumental variables. MR-Egger regression tests for directional pleiotropy by fitting a linear regression model to the causal estimates and their standard errors. The weighted median method provides a robust estimate by taking the median of the causal estimates from the genetic instruments, weighted by their precision.

Network mediation analysis

Network mediation analysis was performed to identify potential mediators of the relationship between PH subtypes and brain structures. This method estimates the direct and indirect effects of the exposure on the outcome through potential mediators. We first scanned for circulation immune cells derived from the OpenGwas platform (https://gwas.mrcieu.ac.uk/) to find potential mediators with significant causal effects from CTEPH or PAH to brain structure using twice two-sample Mendelian randomization. Then, we validated the mediation effects through the effect sizes and standard errors based on network Mendelian randomization [15-17].

Statistical analysis

All analyses were performed with R software, version 4.2.2 (The R Foundation, Vienna, Austria). Mendelian randomization analyses were performed primarily with the use of the TwoSampleMR package (version 0.6.2, developed by Stephen Burgess, Cambridge, United Kingdom) and the MRPRESSO package (version 1.0, developed by Tom Palmer, Bristol, United Kingdom). Gene enrichment used a hypergeometric test, and Benjamini-Hochberg's method was used to control the false-positive rate due to multiple tests. Two-sided p-values of less than 0.05 were considered significant.

Data sharing statement

All data can be accessed publicly through the URLs specified in the methods section.

## Results

Instrumental variable selection

We identified seven SNPs as genetic proxies for CTEPH with F-values greater than 10, indicating that these SNPs avoided weak instrumental variable bias as instrumental variables. Meanwhile, we identified 41 valid instrumental variables for PAH (Table 1).

**Table 1:** Instrumental variable information in this study *P<0.05. Results in this table were derived from a case-control genome-wide association study [13,18]. CTEPH, chronic thromboembolic pulmonary hypertension; PAH, pulmonary arterial hypertension; SNP, single-nucleotide polymorphism; SE, standard error; EAF, effect allele frequency.

Disease	SNP	Effect Allele	Other Allele	SE	P-value	Beta	EAF	Sample Size	R^2^	F	Chromosome	Position	Gene
CTEPH	rs687289	A	G	0.05	<0.001*	0.59	0.66	12266	0.009	115.271	9	1.36E+08	ABO
CTEPH	rs7659024	A	G	0.06	<0.001*	0.47	0.75	12266	0.006	69.166	4	1.56E+08	FGG
CTEPH	rs2289252	T	C	0.05	<0.001*	0.26	0.60	12266	0.003	33.344	4	1.87E+08	F11
CTEPH	rs745849	A	G	0.05	<0.001*	-0.27	0.57	12266	0.002	30.460	20	33572178	MYH7B
CTEPH	rs17202899	C	T	0.09	<0.001*	0.47	0.90	12266	0.002	29.832	6	32434481	HLA-DRA
CTEPH	rs78677622	T	C	0.07	<0.001*	-0.36	0.87	12266	0.002	23.144	10	71196698	TSPAN15
CTEPH	rs2288904	A	G	0.06	<0.001*	-0.27	0.79	12266	0.002	19.864	19	10742170	SLC44A2
PAH	rs2050070	G	A	0.08	<0.001*	-0.35	0.64	316622	<0.001	17.303	1	25178581	RP4-706G24.1
PAH	rs142224712	A	C	0.34	<0.001*	1.42	0.01	316622	<0.001	17.800	1	42158536	GUCA2B
PAH	rs115583922	C	T	0.34	<0.001*	1.43	0.01	316622	<0.001	17.913	1	72730851	RP4-660H19.1,RP4-660H19.2
PAH	rs116489832	C	T	0.22	<0.001*	-0.90	0.06	316622	<0.001	17.237	1	1.03E+08	RP5-936J12.1
PAH	rs62197705	A	G	0.12	<0.001*	0.51	0.09	316622	<0.001	17.833	2	1.87E+08	ZC3H15
PAH	rs13068772	G	T	0.09	<0.001*	0.39	0.22	316622	<0.001	17.911	3	1.32E+08	RP11-517B11.4
PAH	rs13059247	C	G	0.10	<0.001*	-0.43	0.24	316622	<0.001	17.807	3	1.77E+08	LINC00578
PAH	rs74505239	C	G	0.13	<0.001*	0.52	0.09	316622	<0.001	16.483	4	8107443	ABLIM2
PAH	rs10066505	A	G	0.11	<0.001*	0.46	0.13	316622	<0.001	17.342	5	1.24E+08	LINC01170
PAH	rs55895438	C	T	0.19	<0.001*	0.86	0.03	316622	<0.001	20.207	6	30615946	PPP1R10
PAH	rs56821023	G	A	0.08	<0.001*	0.35	0.38	316622	<0.001	17.804	6	1.39E+08	RP11-445F6.2
PAH	rs55716205	A	C	0.09	<0.001*	-0.38	0.72	316622	<0.001	20.038	6	1.58E+08	SNX9
PAH	rs12666086	G	T	0.08	<0.001*	-0.36	0.68	316622	<0.001	18.910	7	1185888	ZFAND2A-DT
PAH	rs6978470	C	T	0.09	<0.001*	0.37	0.24	316622	<0.001	17.544	7	14131178	DGKB
PAH	rs116997068	T	G	0.32	<0.001*	1.34	0.01	316622	<0.001	17.535	7	24911277	OSBPL3
PAH	rs6955683	C	T	0.33	<0.001*	1.45	0.96	316622	<0.001	18.846	7	81557568	AC010091.1
PAH	rs141076809	A	G	0.33	<0.001*	1.41	0.01	316622	<0.001	17.661	8	22383652	SLC39A14
PAH	rs185548465	A	G	0.20	<0.001*	0.90	0.04	316622	<0.001	21.034	8	1.45E+08	ARHGAP39
PAH	rs68147517	T	C	0.12	<0.001*	0.47	0.11	316622	<0.001	16.728	9	30090622	ME2P1
PAH	rs7079564	C	T	0.08	<0.001*	0.35	0.45	316622	<0.001	17.840	10	1066326	WDR37
PAH	rs145221984	T	C	0.34	<0.001*	1.41	0.01	316622	<0.001	17.713	10	8375042	RP11-543F8.2
PAH	rs72856766	C	T	0.14	<0.001*	0.57	0.07	316622	<0.001	17.360	10	1.32E+08	STK32C
PAH	rs59607164	G	A	0.13	<0.001*	0.54	0.07	316622	<0.001	16.507	11	1.18E+08	DSCAML1
PAH	rs73084354	G	A	0.12	<0.001*	-0.50	0.17	316622	<0.001	16.656	12	27647471	PPFIBP1
PAH	rs144159880	C	T	0.70	<0.001*	2.89	0.00	316622	<0.001	17.276	12	1.21E+08	CAMKK2
PAH	rs9510483	T	C	0.09	<0.001*	0.36	0.28	316622	<0.001	17.119	13	22981200	RP11-124N19.4
PAH	rs9592653	C	G	0.13	<0.001*	-0.58	0.16	316622	<0.001	21.417	13	69749704	KLHL1
PAH	rs9590282	C	T	0.09	<0.001*	0.39	0.21	316622	<0.001	17.249	13	95480236	CLDN10,CLDN10-AS1
PAH	rs79642892	A	G	0.19	<0.001*	0.79	0.03	316622	<0.001	16.967	15	23470132	GOLGA6L2
PAH	rs2672691	G	C	0.12	<0.001*	-0.49	0.90	316622	<0.001	17.059	15	29288334	FAM189A1
PAH	rs117239581	A	C	0.27	<0.001*	1.12	0.01	316622	<0.001	16.757	15	38116455	RP11-346D14.1
PAH	rs3826154	G	A	0.13	<0.001*	-0.56	0.15	316622	<0.001	18.784	16	69696652	NFAT5
PAH	rs139913752	A	G	0.37	<0.001*	1.56	0.00	316622	<0.001	17.689	16	78317536	WWOX
PAH	rs9901245	G	C	0.17	<0.001*	0.71	0.04	316622	<0.001	17.278	17	1258809	AC144836.1
PAH	rs150064101	G	T	0.31	<0.001*	1.32	0.01	316622	<0.001	17.613	17	7917192	RNF227
PAH	rs141926054	T	C	0.18	<0.001*	0.79	0.03	316622	<0.001	19.845	17	33960322	ASIC2,CTC-304I17.6,RP11-17M24.3
PAH	rs12949800	T	A	0.08	<0.001*	-0.34	0.44	316622	<0.001	17.070	17	73649150	SDK2
PAH	rs143202006	G	A	0.50	<0.001*	2.14	0.00	316622	<0.001	18.581	18	25569497	ZNF521
PAH	rs116937793	G	A	0.27	<0.001*	1.11	0.01	316622	<0.001	17.027	19	15856586	CLEC4O
PAH	rs235370	C	A	0.15	<0.001*	-0.63	0.11	316622	<0.001	16.495	21	44870645	PTTG1IP
PAH	rs9625243	A	C	0.09	<0.001*	-0.40	0.75	316622	<0.001	20.553	22	27484529	RP11-46E17.6

Impacts of CTEPH and PAH on the brain structure and electrical activity

At the global level, there was no significant association between CTEPH and either full cortical surface area (IVW p=0.586) or thickness (IVW p=0.204) (Table 2). Moreover, PAH did not show a significant effect on full cortical surface area or thickness either (Table 3).

**Table 2:** Mendelian randomization estimates from CTEPH on genetical brain structure *P<0.05. All surfaces or thicknesses can be measured, regardless of whether the global surface or thickness is adjusted to highlight specific regional changes. CTEPH, Chronic thromboembolic pulmonary hypertension; IVW, Inverse-variance weighted; MR, Two-sample Mendelian randomization; CI, Confidence interval; PRESSO, Mendelian randomization pleiotropy residual sum and outlier; alphaCz: Alpha power at the vertex electrode; alphaOcc: Alpha power at the occipital; betaCz: Beta power at the vertex electrode; deltaCz: Delta power at the vertex electrode; peakOcc: Alpha peak at the occipital; thetaCz: Theta power at the vertex electrode.

Outcome	IVW-Derived P-value	OR/β (95% CI)	Cochran's Q-derived P-value	MR Egger Intercept-Derived P-value	Global Test Derived From MR PRESSO
alphaCz	0.911	0.00 (-0.04, 0.03)	0.643	0.675	0.706
alphaOcc	0.920	0.00 (-0.04, 0.03)	0.518	0.476	0.580
betaCz	0.966	0.00 (-0.04, 0.03)	0.771	0.499	0.826
deltaCz	0.191	-0.02 (-0.06, 0.01)	0.996	0.984	0.998
peakOcc	0.023*	0.04 (0.01, 0.08)	0.370	0.353	0.434
thetaCz	0.363	-0.02 (-0.05, 0.02)	0.616	0.371	0.660
hippocampal volume	0.715	0.00 (-0.02, 0.01)	0.275	0.090	0.292
intracranial volume	0.125	-0.01 (-0.03, 0.00)	0.137	0.530	0.212
accumbens volume restricted	0.811	0.00 (-0.03, 0.04)	0.002*	0.166	0.022*
accumbens volume unrestricted	0.649	0.01 (-0.03, 0.05)	0.003*	0.190	0.037*
amygdala volume restricted	0.379	0.01 (-0.02, 0.05)	0.003*	0.442	0.033*
amygdala volume unrestricted	0.369	0.02 (-0.02, 0.06)	0.001*	0.495	0.031*
brainstem volume restricted	0.795	0.00 (-0.02, 0.03)	0.156	0.332	0.261
brainstem volume unrestricted	0.658	0.01 (-0.02, 0.04)	0.053	0.251	0.114
caudate volume restricted	0.503	0.01 (-0.01, 0.02)	0.604	0.189	0.559
caudate volume unrestricted	0.381	0.01 (-0.01, 0.03)	0.364	0.112	0.398
pallidum restricted	0.765	0.00 (-0.02, 0.02)	0.223	0.780	0.327
pallidum unrestricted	0.438	0.01 (-0.01, 0.03)	0.225	0.661	0.364
putamen restricted	0.558	0.01 (-0.02, 0.04)	0.006*	0.759	0.041*
putamen unrestricted	0.568	0.01 (-0.02, 0.05)	0.004*	0.587	0.036*
thalamus volume restricted	0.444	0.01 (-0.01, 0.02)	0.877	0.940	0.869
thalamus volume unrestricted	0.392	0.01 (-0.01, 0.03)	0.739	0.811	0.711
amygdala volume change rate from meta analysis	0.182	0.88 (-0.41, 2.17)	0.333	0.156	0.447
caudate volume change rate from meta analysis	0.758	-0.26 (-1.95, 1.42)	0.421	0.898	0.509
cerebellum grey matter volume change rate from meta analysis	0.663	-4.63 (-25.46, 16.21)	0.340	0.386	0.443
cerebellum white matter volume change rate from meta analysis	0.916	0.41 (-7.23, 8.05)	0.532	0.834	0.461
cerebral white matter volume change rate from meta analysis	0.921	-2.91 (-60.38, 54.55)	0.776	0.435	0.661
cortical grey matter volume change rate from meta analysis	0.634	18.57 (-57.92, 95.07)	0.587	0.130	0.499
hippocampus volume change rate from meta analysis	0.525	0.87 (-1.80, 3.54)	0.052	0.152	0.102
lateral ventricles volume change rate from meta analysis	0.937	0.51 (-12.16, 13.19)	0.404	0.076	0.384
mean thickness from meta analysis	0.452	0.14 (-0.23, 0.51)	0.663	0.164	0.621
nucleus accumbens volume change rate from meta analysis	0.923	-0.03 (-0.64, 0.58)	0.961	0.472	0.966
pallidum volume change rate from meta analysis	0.951	0.04 (-1.34, 1.43)	0.295	0.690	0.381
putamen volume change rate from meta analysis	0.120	-2.03 (-4.58, 0.53)	0.946	0.495	0.926
surface area from meta analysis	0.122	-11.27 (-25.57, 3.02)	0.929	0.420	0.933
thalamus volume change rate from meta analysis	0.395	-1.17 (-3.85, 1.52)	0.935	0.749	0.946
total brain volume change rate from meta analysis	0.495	42.07 (-78.87, 163.01)	0.878	0.545	0.808
amygdala volume change rate from meta regression using linear change	0.813	0.49 (-3.59, 4.58)	0.124	0.930	0.239
caudate volume change rate from meta regression using linear change	0.346	-2.65 (-8.17, 2.86)	0.039*	0.502	0.090
cerebellum grey matter volume change rate from meta regression using linear change	0.695	-9.53 (-57.13, 38.08)	0.432	0.931	0.489
cerebellum white matter volume change rate from meta regression using linear change	0.561	-5.52 (-24.14, 13.09)	0.964	0.699	0.963
cerebral white matter volume change rate from meta regression using linear change	0.592	-35.28 (-164.41, 93.85)	0.713	0.648	0.711
cortical grey matter volume change rate from meta regression using linear change	0.495	-114.13 (-441.98, 213.71)	0.202	0.674	0.264
hippocampus volume change rate from meta regression using linear change	0.920	-0.35 (-7.13, 6.43)	0.015*	0.156	0.048*
lateral ventricles volume change rate from meta regression using linear change	0.039*	22.56 (1.15, 43.97)	0.798	0.801	0.830
mean thickness from meta regression using linear change	0.241	-0.72 (-1.92, 0.48)	0.641	0.876	0.664
nucleus accumbens volume change rate from meta regression using linear change	0.712	-0.31 (-1.94, 1.33)	0.500	0.190	0.586
pallidum volume change rate from meta regression using linear change	0.417	-1.42 (-4.84, 2.01)	0.525	0.373	0.443
putamen volume change rate from meta regression using linear change	0.861	0.54 (-5.52, 6.60)	0.866	0.342	0.873
surface area from meta regression using linear change	0.209	-27.64 (-70.81, 15.52)	0.264	0.780	0.330
thalamus volume change rate from meta regression using linear change	0.025*	-7.64 (-14.31, -0.97)	0.697	0.921	0.761
total brain volume change rate from meta regression using linear change	0.473	-141.77 (-529.31, 245.77)	0.296	0.684	0.349
amygdala volume change rate from meta regression using quadratic change	0.714	1.04 (-4.54, 6.63)	0.430	0.226	0.507
caudate volume change rate from meta regression using quadratic change	0.309	-3.58 (-10.46, 3.31)	0.710	0.132	0.644
cerebellum grey matter volume change rate from meta regression using quadratic change	0.396	-54.32 (-179.61, 70.98)	0.019*	0.574	0.060
cerebellum white matter volume change rate from meta regression using quadratic change	0.262	-23.78 (-65.31, 17.75)	0.260	0.086	0.311
cerebral white matter volume change rate from meta regression using quadratic change	0.664	-63.20 (-348.62, 222.23)	0.128	0.933	0.177
cortical grey matter volume change rate from meta regression using quadratic change	0.521	-143.24 (-580.81, 294.33)	0.745	0.987	0.751
hippocampus volume change rate from meta regression using quadratic change	0.951	0.36 (-11.00, 11.71)	0.028*	0.485	0.088
lateral ventricles volume change rate from meta regression using quadratic change	0.151	32.10 (-11.69, 75.88)	0.505	0.871	0.598
mean thickness from meta regression using quadratic change	0.168	-1.37 (-3.31, 0.58)	0.975	0.602	0.976
nucleus accumbens volume change rate from meta regression using quadratic change	0.857	-0.33 (-3.89, 3.24)	0.161	0.144	0.201
pallidum volume change rate from meta regression using quadratic change	0.904	-0.40 (-7.00, 6.19)	0.348	0.450	0.440
putamen volume change rate from meta regression using quadratic change	0.764	-2.20 (-16.57, 12.16)	0.116	0.632	0.177
surface area from meta regression using quadratic change	0.847	8.20 (-75.24, 91.64)	0.126	0.740	0.184
thalamus volume change rate from meta regression using quadratic change	0.127	-9.15 (-20.90, 2.61)	0.795	0.637	0.833
total brain volume change rate from meta regression using quadratic change	0.187	-446.15 (-1109.33, 217.03)	0.225	0.966	0.324
bankssts surface	0.869	0.31 (-3.32, 3.93)	0.046*	0.742	0.090
bankssts thickness	0.821	0.00 (0.00, 0.01)	0.005*	0.983	0.041*
caudalanteriorcingulate surface	0.809	0.37 (-2.62, 3.36)	0.082	0.750	0.160
caudalanteriorcingulate thickness	0.094	0.00 (-0.01, 0.00)	0.740	0.896	0.662
caudalmiddlefrontal surface	0.986	0.06 (-6.50, 6.62)	0.317	0.679	0.406
caudalmiddlefrontal thickness	0.009*	0.00 (-0.01, 0.00)	0.332	0.166	0.288
cuneus surface	0.113	-2.90 (-6.49, 0.69)	0.323	0.844	0.452
cuneus thickness	0.541	0.00 (0.00, 0.00)	0.909	0.955	0.906
entorhinal surface	0.406	0.93 (-1.26, 3.11)	0.008*	0.964	0.045*
entorhinal thickness	0.257	0.00 (-0.01, 0.00)	0.737	0.316	0.772
frontalpole surface	0.447	-0.34 (-1.21, 0.53)	0.042*	0.644	0.116
frontalpole thickness	0.919	0.00 (0.00, 0.00)	0.413	0.185	0.420
Full surface	0.586	-135.59 (-623.98, 352.79)	0.002*	0.963	0.017*
Full thickness	0.204	0.00 (0.00, 0.00)	0.474	0.316	0.563
fusiform surface	0.538	-3.56 (-14.91, 7.78)	0.008*	0.966	0.048*
fusiform thickness	0.926	0.00 (0.00, 0.00)	0.228	0.444	0.284
inferiorparietal surface	0.750	3.21 (-16.55, 22.98)	0.005*	0.953	0.025*
inferiorparietal thickness	0.345	0.00 (0.00, 0.00)	0.810	0.552	0.864
inferiortemporal surface	0.844	-1.46 (-15.99, 13.08)	0.001*	0.933	0.013*
inferiortemporal thickness	0.764	0.00 (0.00, 0.00)	0.761	0.251	0.811
insula surface	0.271	-2.63 (-7.31, 2.05)	0.272	0.701	0.370
insula thickness	0.622	0.00 (0.00, 0.00)	0.048*	0.089	0.102
isthmuscingulate surface	0.400	-1.27 (-4.21, 1.68)	0.276	0.883	0.348
isthmuscingulate thickness	0.872	0.00 (0.00, 0.00)	0.428	0.792	0.533
lateraloccipital surface	0.340	-6.61 (-20.20, 6.98)	0.055	0.851	0.151
lateraloccipital thickness	0.380	0.00 (0.00, 0.00)	0.386	0.495	0.484
lateralorbitofrontal surface	0.477	-3.25 (-12.21, 5.71)	0.005*	0.821	0.044*
lateralorbitofrontal thickness	0.271	0.00 (0.00, 0.00)	0.518	0.137	0.458
lingual surface	0.152	-4.83 (-11.44, 1.78)	0.613	0.619	0.611
lingual thickness	0.389	0.00 (0.00, 0.00)	0.711	0.764	0.752
medialorbitofrontal surface	0.520	-2.52 (-10.22, 5.17)	0.000*	0.951	0.005*
medialorbitofrontal thickness	0.157	0.00 (0.00, 0.00)	0.863	0.400	0.873
middletemporal surface	0.652	-2.50 (-13.35, 8.36)	0.023*	0.692	0.062
middletemporal thickness	0.853	0.00 (0.00, 0.00)	0.737	0.442	0.808
paracentral surface	0.437	-1.83 (-6.44, 2.78)	0.082	0.454	0.165
paracentral thickness	0.030*	0.00 (-0.01, 0.00)	0.310	0.214	0.333
parahippocampal surface	0.382	-1.04 (-3.36, 1.29)	0.071	0.752	0.142
parahippocampal thickness	0.982	0.00 (-0.01, 0.01)	0.002*	0.765	0.024*
parsopercularis surface	0.721	0.95 (-4.27, 6.17)	0.085	0.973	0.172
parsopercularis thickness	0.707	0.00 (0.00, 0.00)	0.381	0.681	0.396
parsorbitalis surface	0.529	-0.95 (-3.91, 2.01)	0.001*	0.605	0.014*
parsorbitalis thickness	0.213	0.00 (-0.01, 0.00)	0.072	0.060	0.129
parstriangularis surface	0.928	0.24 (-4.96, 5.44)	0.033*	0.979	0.101
parstriangularis thickness	0.758	0.00 (0.00, 0.00)	0.367	0.285	0.466
pericalcarine surface	0.343	-2.03 (-6.21, 2.16)	0.364	0.654	0.494
pericalcarine thickness	0.320	0.00 (0.00, 0.00)	0.354	0.785	0.416
postcentral surface	0.545	-3.69 (-15.63, 8.26)	0.042*	0.665	0.138
postcentral thickness	0.941	0.00 (0.00, 0.00)	0.573	0.584	0.592
posteriorcingulate surface	0.527	-1.70 (-6.99, 3.58)	0.002*	0.935	0.015*
posteriorcingulate thickness	0.183	0.00 (-0.01, 0.00)	0.155	0.094	0.205
precentral surface	0.914	-0.74 (-14.24, 12.75)	0.031*	0.448	0.091
precentral thickness	0.532	0.00 (0.00, 0.00)	0.438	0.304	0.459
precuneus surface	0.202	-10.79 (-27.35, 5.78)	0.000*	0.630	0.001*
precuneus thickness	0.683	0.00 (0.00, 0.00)	0.950	0.807	0.939
rostralanteriorcingulate surface	0.339	-2.30 (-7.00, 2.41)	0.001*	0.736	0.016*
rostralanteriorcingulate thickness	0.322	0.00 (0.00, 0.01)	0.661	0.659	0.781
rostralmiddlefrontal surface	0.459	-8.05 (-29.35, 13.25)	0.009*	0.864	0.043*
rostralmiddlefrontal thickness	0.134	0.00 (0.00, 0.00)	0.742	0.631	0.684
superiorfrontal surface	0.412	-8.42 (-28.55, 11.71)	0.042*	0.658	0.123
superiorfrontal thickness	0.103	0.00 (0.00, 0.00)	0.394	0.208	0.392
superiorparietal surface	0.111	-9.31 (-20.77, 2.15)	0.296	0.557	0.366
superiorparietal thickness	0.043*	0.00 (0.00, 0.00)	0.998	0.972	0.998
superiortemporal surface	0.287	-6.77 (-19.24, 5.70)	0.006*	0.920	0.042*
superiortemporal thickness	0.798	0.00 (0.00, 0.00)	0.086	0.731	0.175
supramarginal surface	0.704	-3.37 (-20.78, 14.04)	0.001*	0.921	0.011*
supramarginal thickness	0.080	0.00 (0.00, 0.00)	0.650	0.332	0.660
temporalpole surface	0.901	-0.09 (-1.46, 1.29)	0.047*	0.368	0.114
temporalpole thickness	0.400	0.00 (-0.01, 0.00)	0.362	0.134	0.408
transversetemporal surface	0.604	-0.39 (-1.88, 1.09)	0.098	0.798	0.178
transversetemporal thickness	0.216	0.00 (-0.01, 0.00)	0.244	0.105	0.240
bankssts surface with global surface weight	0.152	1.69 (-0.62, 4.01)	0.220	0.725	0.342
caudalanteriorcingulate surface with global surface weight	0.116	1.40 (-0.34, 3.14)	0.876	0.406	0.892
caudalmiddlefrontal surface with global surface weight	0.528	1.92 (-4.06, 7.91)	0.133	0.712	0.191
cuneus surface with global surface weight	0.672	-0.57 (-3.21, 2.07)	0.528	0.876	0.614
entorhinal surface with global surface weight	0.047*	1.13 (0.01, 2.25)	0.405	0.800	0.472
frontalpole surface with global surface weight	0.850	-0.06 (-0.65, 0.54)	0.257	0.340	0.290
fusiform surface with global surface weight	0.788	-0.60 (-4.98, 3.78)	0.872	0.746	0.901
inferiorparietal surface with global surface weight	0.022*	10.98 (1.59, 20.37)	0.162	0.776	0.269
inferiortemporal surface with global surface weight	0.419	2.21 (-3.15, 7.58)	0.324	0.535	0.380
insula surface with global surface weight	0.541	-1.19 (-5.01, 2.63)	0.145	0.490	0.254
isthmuscingulate surface with global surface weight	0.997	0.00 (-2.16, 2.15)	0.326	0.807	0.390
lateraloccipital surface with global surface weight	0.689	-1.35 (-7.94, 5.25)	0.550	0.486	0.607
lateralorbitofrontal surface with global surface weight	0.318	-1.64 (-4.85, 1.58)	0.593	0.559	0.502
lingual surface with global surface weight	0.648	-1.44 (-7.62, 4.74)	0.237	0.581	0.344
medialorbitofrontal surface with global surface weight	0.605	-0.71 (-3.41, 1.99)	0.271	0.956	0.408
middletemporal surface with global surface weight	0.454	1.80 (-2.91, 6.50)	0.304	0.734	0.442
paracentral surface with global surface weight	0.788	-0.36 (-2.95, 2.24)	0.453	0.207	0.453
parahippocampal surface with global surface weight	0.406	-0.57 (-1.91, 0.77)	0.967	0.688	0.962
parsopercularis surface with global surface weight	0.284	1.68 (-1.39, 4.76)	0.679	0.820	0.654
parsorbitalis surface with global surface weight	0.624	-0.34 (-1.71, 1.03)	0.153	0.248	0.222
parstriangularis surface with global surface weight	0.339	1.39 (-1.46, 4.25)	0.567	0.813	0.546
pericalcarine surface with global surface weight	0.890	-0.28 (-4.27, 3.71)	0.264	0.675	0.360
postcentral surface with global surface weight	0.951	0.17 (-5.25, 5.58)	0.311	0.221	0.320
posteriorcingulate surface with global surface weight	0.955	-0.06 (-2.23, 2.11)	0.318	0.848	0.449
precentral surface with global surface weight	0.318	3.77 (-3.63, 11.18)	0.154	0.091	0.204
precuneus surface with global surface weight	0.079	-6.28 (-13.30, 0.73)	0.079	0.187	0.157
rostralanteriorcingulate surface with global surface weight	0.243	-1.20 (-3.21, 0.81)	0.215	0.365	0.328
rostralmiddlefrontal surface with global surface weight	0.369	-3.35 (-10.66, 3.96)	0.367	0.636	0.397
superiorfrontal surface with global surface weight	0.715	-1.31 (-8.34, 5.72)	0.492	0.334	0.415
superiorparietal surface with global surface weight	0.551	-3.07 (-13.14, 7.01)	0.076	0.545	0.155
superiortemporal surface with global surface weight	0.501	-1.83 (-7.17, 3.50)	0.142	0.953	0.252
supramarginal surface with global surface weight	0.820	0.87 (-6.63, 8.36)	0.128	0.895	0.206
temporalpole surface with global surface weight	0.507	0.31 (-0.61, 1.23)	0.307	0.139	0.356
transversetemporal surface with global surface weight	0.947	0.03 (-0.86, 0.92)	0.441	0.691	0.580
bankssts surface with global thickness weight	0.423	0.00 (0.00, 0.01)	0.002*	0.442	0.034*
caudalanteriorcingulate surface with global thickness weight	0.188	0.00 (-0.01, 0.00)	0.656	0.624	0.622
caudalmiddlefrontal surface with global thickness weight	0.016*	0.00 (0.00, 0.00)	0.401	0.353	0.365
cuneus surface with global thickness weight	0.644	0.00 (0.00, 0.00)	0.616	0.419	0.681
entorhinal surface with global thickness weight	0.447	0.00 (-0.01, 0.00)	0.452	0.176	0.519
frontalpole surface with global thickness weight	0.689	0.00 (0.00, 0.01)	0.156	0.057	0.186
fusiform surface with global thickness weight	0.373	0.00 (0.00, 0.00)	0.216	0.896	0.282
inferiorparietal surface with global thickness weight	0.721	0.00 (0.00, 0.00)	0.781	0.914	0.815
inferiortemporal surface with global thickness weight	0.226	0.00 (0.00, 0.00)	0.691	0.422	0.743
insula surface with global thickness weight	0.778	0.00 (0.00, 0.00)	0.038*	0.138	0.094
isthmuscingulate surface with global thickness weight	0.813	0.00 (0.00, 0.00)	0.458	0.438	0.511
lateraloccipital surface with global thickness weight	0.816	0.00 (0.00, 0.00)	0.293	0.829	0.429
lateralorbitofrontal surface with global thickness weight	0.009*	0.00 (0.00, 0.00)	0.603	0.216	0.553
lingual surface with global thickness weight	0.663	0.00 (0.00, 0.00)	0.796	0.664	0.846
medialorbitofrontal surface with global thickness weight	0.021*	0.00 (0.00, 0.01)	0.970	0.660	0.974
middletemporal surface with global thickness weight	0.625	0.00 (0.00, 0.00)	0.162	0.947	0.257
paracentral surface with global thickness weight	0.098	0.00 (0.00, 0.00)	0.200	0.705	0.234
parahippocampal surface with global thickness weight	0.870	0.00 (-0.01, 0.01)	0.003*	0.608	0.041*
parsopercularis surface with global thickness weight	0.576	0.00 (0.00, 0.00)	0.398	0.794	0.429
parsorbitalis surface with global thickness weight	0.252	0.00 (0.00, 0.00)	0.279	0.098	0.359
parstriangularis surface with global thickness weight	0.696	0.00 (0.00, 0.00)	0.667	0.629	0.765
pericalcarine surface with global thickness weight	0.486	0.00 (0.00, 0.00)	0.169	0.478	0.206
postcentral surface with global thickness weight	0.398	0.00 (0.00, 0.00)	0.870	0.557	0.884
posteriorcingulate surface with global thickness weight	0.207	0.00 (0.00, 0.00)	0.216	0.211	0.250
precentral surface with global thickness weight	0.907	0.00 (0.00, 0.00)	0.755	0.831	0.685
precuneus surface with global thickness weight	0.384	0.00 (0.00, 0.00)	0.630	0.218	0.628
rostralanteriorcingulate surface with global thickness weight	0.104	0.00 (0.00, 0.01)	0.534	0.986	0.649
rostralmiddlefrontal surface with global thickness weight	0.533	0.00 (0.00, 0.00)	0.765	0.803	0.765
superiorfrontal surface with global thickness weight	0.306	0.00 (0.00, 0.00)	0.605	0.598	0.532
superiorparietal surface with global thickness weight	0.046*	0.00 (0.00, 0.00)	0.609	0.219	0.604
superiortemporal surface with global thickness weight	0.242	0.00 (0.00, 0.00)	0.083	0.465	0.146
supramarginal surface with global thickness weight	0.249	0.00 (0.00, 0.00)	0.220	0.762	0.330
temporalpole surface with global thickness weight	0.730	0.00 (-0.01, 0.00)	0.322	0.230	0.374
transversetemporal surface with global thickness weight	0.439	0.00 (-0.01, 0.00)	0.181	0.160	0.229

**Table 3:** Mendelian randomization estimates from PAH on genetical brain structure *P<0.05. All surfaces or thicknesses can be measured with or without adjusting the global surface or thickness to highlight specific region changes. PAH, pulmonary arterial hypertension; IVW, inverse-variance weighted; MR, two-sample Mendelian randomization; CI, confidence interval; PRESSO, Mendelian randomization pleiotropy residual sum and outlier; alphaCz: alpha power at the vertex electrode; alphaOcc: alpha power at the occipital; betaCz: beta power at the vertex electrode; deltaCz: delta power at the vertex electrode; peakOcc: alpha peak at the occipital; thetaCz: theta power at the vertex electrode.

Outcome	IVW-Derived P-value	β (95% CI)	Cochran's Q-Derived P-value	MR Egger Intercept-Derived P-value	Global Test Derived From MR PRESSO
alphaCz	0.326	-0.01 (-0.04, 0.01)	0.408	0.142	0.533
alphaOcc	0.253	-0.01 (-0.04, 0.01)	0.724	0.475	0.824
betaCz	0.360	-0.01 (-0.04, 0.01)	0.647	0.458	0.738
deltaCz	0.246	-0.02 (-0.05, 0.01)	0.073	0.608	0.132
peakOcc	0.099	0.03 (-0.01, 0.06)	0.066	0.286	0.151
thetaCz	0.464	-0.01 (-0.04, 0.02)	0.098	0.241	0.124
hippocampal volume	0.048*	0.00 (0.00, 0.01)	0.108	0.741	0.281
intracranial volume	0.702	0.00 (-0.00, 0.01)	0.142	0.991	0.312
accumbens volume restricted	0.555	0.00 (-0.01, 0.02)	0.003*	0.752	0.030*
accumbens volume unrestricted	0.506	0.00 (-0.01, 0.02)	0.010*	0.945	0.048*
amygdala volume restricted	0.118	0.01 (-0.00, 0.02)	0.074	0.495	0.117
amygdala volume unrestricted	0.021*	0.01 (0.00, 0.02)	0.378	0.378	0.435
brainstem volume restricted	0.886	-0.00 (-0.01, 0.01)	0.475	0.842	0.517
brainstem volume unrestricted	0.773	0.00 (-0.01, 0.01)	0.264	0.840	0.356
caudate volume restricted	0.317	0.00 (-0.00, 0.01)	0.571	0.499	0.461
caudate volume unrestricted	0.182	0.01 (-0.00, 0.02)	0.328	0.623	0.218
pallidum restricted	0.415	-0.00 (-0.01, 0.01)	0.473	0.953	0.567
pallidum unrestricted	0.753	-0.00 (-0.01, 0.01)	0.452	0.884	0.491
putamen restricted	0.312	0.01 (-0.01, 0.02)	0.013*	0.575	0.048*
putamen unrestricted	0.155	0.01 (-0.00, 0.03)	0.004*	0.700	0.014*
thalamus volume restricted	0.758	0.00 (-0.01, 0.01)	0.347	0.907	0.251
thalamus volume unrestricted	0.590	0.00 (-0.01, 0.01)	0.322	0.847	0.252
amygdala volume change rate from meta analysis	0.998	0.00 (-0.84, 0.84)	0.658	0.213	0.828
caudate volume change rate from meta analysis	0.736	-0.32 (-2.18, 1.54)	0.001*	0.846	0.007*
cerebellum grey matter volume change rate from meta analysis	0.631	3.37 (-10.38, 17.12)	0.486	0.396	0.408
cerebellum white matter volume change rate from meta analysis	0.332	-2.99 (-9.03, 3.05)	0.237	0.197	0.124
cerebral white matter volume change rate from meta analysis	0.033	-52.72 (-101.21, -4.24)	0.121	0.496	0.186
cortical grey matter volume change rate from meta analysis	0.305	39.31(-35.82, 114.44)	0.014*	0.877	0.005*
hippocampus volume change rate from meta analysis	0.183	-0.91 (-2.26, 0.43)	0.388	0.166	0.583
lateral ventricles volume change rate from meta analysis	0.236	-5.43 (-14.42, 3.56)	0.508	0.272	0.210
mean thickness from meta analysis	0.457	0.14 (-0.23, 0.52)	0.008*	0.586	0.001*
nucleus accumbens volume change rate from meta analysis	0.496	-0.15 (-0.57, 0.28)	0.786	0.575	0.775
pallidum volume change rate from meta analysis	0.692	-0.18 (-1.06, 0.71)	0.423	0.291	0.461
putamen volume change rate from meta analysis	0.182	1.45 (-0.68, 3.59)	0.118	0.710	0.083
surface area from meta analysis	0.653	-2.49 (-13.35, 8.37)	0.263	0.433	0.151
thalamus volume change rate from meta analysis	0.285	1.01 (-0.85, 2.87)	0.753	0.580	0.665
total brain volume change rate from meta analysis	0.600	23.31 (-63.85, 110.46)	0.385	0.319	0.175
amygdala volume change rate from meta regression using linear change	0.593	0.62 (-1.66, 2.91)	0.757	0.264	0.573
caudate volume change rate from meta regression using linear change	0.925	-0.15 (-3.15, 2.86)	0.245	0.527	0.417
cerebellum grey matter volume change rate from meta regression using linear change	0.780	-6.99 (-55.94, 41.97)	0.022*	0.641	0.060
cerebellum white matter volume change rate from meta regression using linear change	0.999	-0.01 (-13.63, 13.62)	0.818	0.139	0.895
cerebral white matter volume change rate from meta regression using linear change	0.100	-81.00 (-177.49, 15.49)	0.581	0.242	0.618
cortical grey matter volume change rate from meta regression using linear change	0.939	10.06 (-245.98, 266.10)	0.086	0.590	0.095
hippocampus volume change rate from meta regression using linear change	0.055	-3.09 (-6.24, 0.06)	0.391	0.751	0.363
lateral ventricles volume change rate from meta regression using linear change	0.347	-7.70 (-23.75, 8.36)	0.633	0.595	0.506
mean thickness from meta regression using linear change	0.663	0.30 (-1.04, 1.64)	0.006*	0.836	0.012*
nucleus accumbens volume change rate from meta regression using linear change	0.695	-0.27 (-1.63, 1.09)	0.198	0.464	0.313
pallidum volume change rate from meta regression using linear change	0.984	-0.03 (-2.52, 2.47)	0.971	0.604	0.975
putamen volume change rate from meta regression using linear change	0.877	0.48 (-5.57, 6.53)	0.023*	0.739	0.006*
surface area from meta regression using linear change	0.643	6.80 (-21.98, 35.58)	0.378	0.364	0.378
thalamus volume change rate from meta regression using linear change	0.930	0.22 (-4.61, 5.04)	0.944	0.317	0.901
total brain volume change rate from meta regression using linear change	0.928	-14.83 (-338.48, 308.82)	0.105	0.689	0.170
amygdala volume change rate from meta regression using quadratic change	0.564	1.39 (-3.33, 6.11)	0.150	0.772	0.082
caudate volume change rate from meta regression using quadratic change	0.176	3.44 (-1.54, 8.42)	0.673	0.290	0.727
cerebellum grey matter volume change rate from meta regression using quadratic change	0.459	-21.98 (-80.18, 36.22)	0.839	0.487	0.922
cerebellum white matter volume change rate from meta regression using quadratic change	0.720	-4.76 (-30.76, 21.24)	0.772	0.373	0.641
cerebral white matter volume change rate from meta regression using quadratic change	0.702	-45.17 (-276.50, 186.15)	0.020*	0.147	0.059
cortical grey matter volume change rate from meta regression using quadratic change	0.601	105.23 (-289.04, 499.50)	0.122	0.188	0.263
hippocampus volume change rate from meta regression using quadratic change	0.087	-4.71 (-10.09, 0.68)	0.579	0.692	0.758
lateral ventricles volume change rate from meta regression using quadratic change	0.167	-26.09 (-63.11, 10.92)	0.234	0.598	0.087
mean thickness from meta regression using quadratic change	0.361	0.88 (-1.00, 2.76)	0.057	0.546	0.110
nucleus accumbens volume change rate from meta regression using quadratic change	0.368	-0.95 (-3.00, 1.11)	0.969	0.821	0.994
pallidum volume change rate from meta regression using quadratic change	0.715	0.83 (-3.64, 5.30)	0.735	0.241	0.779
putamen volume change rate from meta regression using quadratic change	0.549	2.96 (-6.72, 12.63)	0.097	0.218	0.050
surface area from meta regression using quadratic change	0.369	25.81 (-30.45, 82.06)	0.128	0.082	0.178
thalamus volume change rate from meta regression using quadratic change	0.225	5.20 (-3.19, 13.60)	0.790	0.765	0.539
total brain volume change rate from meta regression using quadratic change	0.884	38.53 (-477.26, 554.32)	0.116	0.132	0.203
bankssts surface	0.146	0.95 (-0.33, 2.23)	0.824	0.462	0.792
bankssts thickness	0.733	0.00 (-0.00, 0.00)	0.623	0.598	0.706
caudalanteriorcingulate surface	0.769	0.17 (-0.96, 1.30)	0.428	0.728	0.549
caudalanteriorcingulate thickness	0.947	0.00 (-0.00, 0.00)	0.048*	0.765	0.150
caudalmiddlefrontal surface	0.329	2.17 (-2.19, 6.52)	0.007*	0.769	0.011*
caudalmiddlefrontal thickness	0.231	0.00 (-0.00, 0.00)	0.061	0.686	0.120
cuneus surface	0.080	1.56 (-0.19, 3.30)	0.825	0.811	0.879
cuneus thickness	0.640	0.00 (-0.00, 0.00)	0.048*	0.942	0.114
entorhinal surface	0.959	0.02 (-0.69, 0.73)	0.236	0.603	0.427
entorhinal thickness	0.070	-0.00 (-0.01, 0.00)	0.462	0.347	0.404
frontalpole surface	0.239	0.20 (-0.13, 0.53)	0.209	0.135	0.391
frontalpole thickness	0.566	0.00 (-0.00, 0.00)	0.810	0.436	0.659
Full surface	0.216	90.96 (-53.29, 235.20)	0.264	0.502	0.450
Full thickness	0.226	0.00 (-0.00, 0.00)	0.273	0.939	0.472
fusiform surface	0.297	1.81 (-1.59, 5.22)	0.620	0.859	0.785
fusiform thickness	0.838	0.00 (-0.00, 0.00)	0.778	0.785	0.784
inferiorparietal surface	0.682	1.18 (-4.45, 6.80)	0.671	0.717	0.675
inferiorparietal thickness	0.102	0.00 (-0.00, 0.00)	0.934	0.830	0.920
inferiortemporal surface	0.157	3.39 (-1.30, 8.08)	0.050	0.809	0.105
inferiortemporal thickness	0.381	0.00 (-0.00, 0.00)	0.587	0.280	0.447
insula surface	0.306	1.15 (-1.06, 3.37)	0.430	0.337	0.471
insula thickness	0.997	0.00 (-0.00, 0.00)	0.634	0.380	0.756
isthmuscingulate surface	0.202	1.00 (-0.54, 2.54)	0.190	0.058	0.375
isthmuscingulate thickness	0.484	0.00 (-0.00, 0.00)	0.090	0.595	0.114
lateraloccipital surface	0.373	2.50 (-3.00, 8.01)	0.154	0.401	0.345
lateraloccipital thickness	0.638	0.00 (-0.00, 0.00)	0.557	0.991	0.799
lateralorbitofrontal surface	0.459	0.97 (-1.59, 3.52)	0.644	0.927	0.808
lateralorbitofrontal thickness	0.490	0.00 (-0.00, 0.00)	0.488	0.150	0.538
lingual surface	0.181	2.36 (-1.10, 5.83)	0.793	0.685	0.925
lingual thickness	0.509	0.00 (-0.00, 0.00)	0.983	0.377	0.985
medialorbitofrontal surface	0.425	0.89 (-1.29, 3.06)	0.146	0.909	0.285
medialorbitofrontal thickness	0.877	-0.00 (-0.00, 0.00)	0.028*	0.280	0.121
middletemporal surface	0.545	1.20 (-2.69, 5.10)	0.238	0.695	0.390
middletemporal thickness	0.237	0.00 (-0.00, 0.00)	0.320	0.895	0.350
paracentral surface	0.097	1.73 (-0.32, 3.78)	0.098	0.412	0.122
paracentral thickness	0.475	0.00 (-0.00, 0.00)	0.132	0.803	0.076
parahippocampal surface	0.491	0.34 (-0.63, 1.30)	0.248	0.687	0.145
parahippocampal thickness	0.830	-0.00 (-0.00, 0.00)	0.934	0.672	0.570
parsopercularis surface	0.749	0.32 (-1.64, 2.28)	0.705	0.629	0.686
parsopercularis thickness	0.111	0.00 (-0.00, 0.00)	0.762	0.268	0.690
parsorbitalis surface	0.716	0.14 (-0.60, 0.87)	0.568	0.855	0.721
parsorbitalis thickness	0.758	0.00 (-0.00, 0.00)	0.293	0.715	0.340
parstriangularis surface	0.983	-0.02 (-1.76, 1.72)	0.466	0.844	0.656
parstriangularis thickness	0.248	0.00 (-0.00, 0.00)	0.525	0.292	0.634
pericalcarine surface	0.272	1.26 (-0.99, 3.51)	0.288	0.933	0.342
pericalcarine thickness	0.268	0.00 (-0.00, 0.00)	0.631	0.137	0.708
postcentral surface	0.099	3.52 (-0.66, 7.70)	0.693	0.710	0.873
postcentral thickness	0.403	0.00 (-0.00, 0.00)	0.746	0.801	0.836
posteriorcingulate surface	0.819	0.17 (-1.26, 1.59)	0.709	0.657	0.803
posteriorcingulate thickness	0.050	0.00 (-0.00, 0.00)	0.522	0.646	0.597
precentral surface	0.316	2.63 (-2.52, 7.77)	0.153	0.098	0.217
precentral thickness	0.741	0.00 (-0.00, 0.00)	0.248	0.893	0.385
precuneus surface	0.128	3.63 (-1.04, 8.29)	0.167	0.242	0.291
precuneus thickness	0.114	0.00 (-0.00, 0.00)	0.202	0.963	0.339
rostralanteriorcingulate surface	0.429	0.55 (-0.82, 1.92)	0.158	0.825	0.324
rostralanteriorcingulate thickness	0.697	-0.00 (-0.00, 0.00)	0.021*	0.010*	0.059
rostralmiddlefrontal surface	0.675	1.36 (-4.99, 7.71)	0.831	0.327	0.926
rostralmiddlefrontal thickness	0.233	0.00 (-0.00, 0.00)	0.774	0.500	0.900
superiorfrontal surface	0.473	2.84 (-4.90, 10.58)	0.195	0.442	0.372
superiorfrontal thickness	0.995	-0.00 (-0.00, 0.00)	0.064	0.273	0.106
superiorparietal surface	0.500	1.87 (-3.57, 7.31)	0.479	0.406	0.729
superiorparietal thickness	0.195	0.00 (-0.00, 0.00)	0.298	0.843	0.322
superiortemporal surface	0.981	0.05 (-4.14, 4.24)	0.159	0.794	0.317
superiortemporal thickness	0.161	0.00 (-0.00, 0.00)	0.226	0.458	0.110
supramarginal surface	0.961	-0.14 (-5.89, 5.60)	0.034*	0.621	0.076
supramarginal thickness	0.216	0.00 (-0.00, 0.00)	0.863	0.559	0.944
temporalpole surface	0.928	0.02 (-0.46, 0.51)	0.422	0.680	0.554
temporalpole thickness	0.866	0.00 (-0.00, 0.00)	0.021*	0.202	0.061
transversetemporal surface	0.980	0.01 (-0.68, 0.70)	0.074	0.878	0.161
transversetemporal thickness	0.518	0.00 (-0.00, 0.00)	0.611	0.837	0.688
bankssts surface with global surface weight	0.395	0.48 (-0.63, 1.59)	0.284	0.093	0.316
caudalanteriorcingulate surface with global surface weight	0.543	-0.28 (-1.19, 0.63)	0.574	0.269	0.576
caudalmiddlefrontal surface with global surface weight	0.715	0.63 (-2.74, 3.99)	0.005*	0.913	0.011*
cuneus surface with global surface weight	0.225	0.86 (-0.53, 2.24)	0.650	0.712	0.780
entorhinal surface with global surface weight	0.623	-0.16 (-0.79, 0.47)	0.265	0.755	0.398
frontalpole surface with global surface weight	0.594	0.09 (-0.24, 0.41)	0.082	0.260	0.132
fusiform surface with global surface weight	0.705	-0.47 (-2.92, 1.98)	0.309	0.869	0.439
inferiorparietal surface with global surface weight	0.646	-1.10 (-5.81, 3.60)	0.076	0.921	0.105
inferiortemporal surface with global surface weight	0.357	1.50 (-1.69, 4.70)	0.057	0.798	0.085
insula surface with global surface weight	0.593	0.43 (-1.14, 2.00)	0.851	0.494	0.800
isthmuscingulate surface with global surface weight	0.504	0.39 (-0.76, 1.55)	0.245	0.066	0.354
lateraloccipital surface with global surface weight	0.774	0.52 (-3.02, 4.05)	0.388	0.766	0.628
lateralorbitofrontal surface with global surface weight	0.612	-0.43 (-2.10, 1.24)	0.638	0.355	0.824
lingual surface with global surface weight	0.599	0.74 (-2.03, 3.51)	0.556	0.848	0.685
medialorbitofrontal surface with global surface weight	0.799	0.19 (-1.24, 1.62)	0.139	0.431	0.214
middletemporal surface with global surface weight	0.682	-0.47 (-2.73, 1.79)	0.509	0.872	0.609
paracentral surface with global surface weight	0.329	0.83 (-0.83, 2.49)	0.064	0.657	0.069
parahippocampal surface with global surface weight	0.940	0.03 (-0.73, 0.79)	0.335	0.987	0.152
parsopercularis surface with global surface weight	0.682	-0.33 (-1.93, 1.27)	0.867	0.216	0.831
parsorbitalis surface with global surface weight	0.648	-0.14 (-0.76, 0.47)	0.254	0.322	0.344
parstriangularis surface with global surface weight	0.520	-0.49 (-1.97, 1.00)	0.602	0.512	0.830
pericalcarine surface with global surface weight	0.510	0.70 (-1.39, 2.80)	0.157	0.921	0.165
postcentral surface with global surface weight	0.214	1.67 (-0.96, 4.30)	0.882	0.746	0.918
posteriorcingulate surface with global surface weight	0.303	-0.55 (-1.60, 0.50)	0.704	0.764	0.771
precentral surface with global surface weight	0.875	0.24 (-2.81, 3.30)	0.601	0.061	0.807
precuneus surface with global surface weight	0.126	2.12 (-0.60, 4.84)	0.350	0.381	0.476
rostralanteriorcingulate surface with global surface weight	0.801	0.13 (-0.90, 1.16)	0.118	0.505	0.208
rostralmiddlefrontal surface with global surface weight	0.363	-1.69 (-5.34, 1.95)	0.658	0.584	0.802
superiorfrontal surface with global surface weight	0.532	-1.17 (-4.84, 2.50)	0.486	0.396	0.661
superiorparietal surface with global surface weight	0.789	-0.58 (-4.78, 3.63)	0.174	0.717	0.351
superiortemporal surface with global surface weight	0.272	-1.25 (-3.49, 0.98)	0.401	0.512	0.676
supramarginal surface with global surface weight	0.459	-1.52 (-5.56, 2.51)	0.015*	0.141	0.023*
temporalpole surface with global surface weight	0.693	-0.09 (-0.52, 0.35)	0.444	0.323	0.542
transversetemporal surface with global surface weight	0.488	-0.19 (-0.71, 0.34)	0.179	0.888	0.326
bankssts surface with global thickness weight	0.599	-0.00 (-0.00, 0.00)	0.565	0.972	0.724
caudalanteriorcingulate surface with global thickness weight	0.664	-0.00 (-0.00, 0.00)	0.227	0.863	0.437
caudalmiddlefrontal surface with global thickness weight	0.601	0.00 (-0.00, 0.00)	0.003*	0.538	0.012*
cuneus surface with global thickness weight	0.982	0.00 (-0.00, 0.00)	0.379	0.820	0.621
entorhinal surface with global thickness weight	0.023	-0.00 (-0.01, -0.00)	0.843	0.237	0.684
frontalpole surface with global thickness weight	0.901	-0.00 (-0.00, 0.00)	0.666	0.481	0.309
fusiform surface with global thickness weight	0.282	-0.00 (-0.00, 0.00)	0.526	0.955	0.393
inferiorparietal surface with global thickness weight	0.157	0.00 (-0.00, 0.00)	0.564	0.514	0.420
inferiortemporal surface with global thickness weight	0.679	-0.00 (-0.00, 0.00)	0.074	0.262	0.023*
insula surface with global thickness weight	0.487	-0.00 (-0.00, 0.00)	0.291	0.552	0.474
isthmuscingulate surface with global thickness weight	0.718	0.00 (-0.00, 0.00)	0.074	0.538	0.052
lateraloccipital surface with global thickness weight	0.581	-0.00 (-0.00, 0.00)	0.823	0.690	0.911
lateralorbitofrontal surface with global thickness weight	0.925	0.00 (-0.00, 0.00)	0.507	0.194	0.518
lingual surface with global thickness weight	0.793	0.00 (-0.00, 0.00)	0.763	0.268	0.757
medialorbitofrontal surface with global thickness weight	0.464	-0.00 (-0.00, 0.00)	0.036*	0.236	0.079
middletemporal surface with global thickness weight	0.838	0.00 (-0.00, 0.00)	0.285	0.969	0.416
paracentral surface with global thickness weight	0.814	-0.00 (-0.00, 0.00)	0.104	0.591	0.011*
parahippocampal surface with global thickness weight	0.554	-0.00 (-0.00, 0.00)	0.990	0.528	0.782
parsopercularis surface with global thickness weight	0.358	0.00 (-0.00, 0.00)	0.427	0.229	0.236
parsorbitalis surface with global thickness weight	0.611	-0.00 (-0.00, 0.00)	0.226	0.660	0.319
parstriangularis surface with global thickness weight	0.759	0.00 (-0.00, 0.00)	0.221	0.356	0.425
pericalcarine surface with global thickness weight	0.405	0.00 (-0.00, 0.00)	0.919	0.093	0.961
postcentral surface with global thickness weight	0.764	0.00 (-0.00, 0.00)	0.223	0.534	0.360
posteriorcingulate surface with global thickness weight	0.077	0.00 (-0.00, 0.00)	0.728	0.612	0.715
precentral surface with global thickness weight	0.408	-0.00 (-0.00, 0.00)	0.117	0.548	0.213
precuneus surface with global thickness weight	0.330	0.00 (-0.00, 0.00)	0.219	0.800	0.383
rostralanteriorcingulate surface with global thickness weight	0.451	-0.00 (-0.00, 0.00)	0.096	0.003*	0.131
rostralmiddlefrontal surface with global thickness weight	0.314	0.00 (-0.00, 0.00)	0.928	0.292	0.960
superiorfrontal surface with global thickness weight	0.178	-0.00 (-0.00, 0.00)	0.255	0.248	0.364
superiorparietal surface with global thickness weight	0.520	0.00 (-0.00, 0.00)	0.480	0.861	0.491
superiortemporal surface with global thickness weight	0.165	0.00 (-0.00, 0.00)	0.519	0.438	0.231
supramarginal surface with global thickness weight	0.185	0.00 (-0.00, 0.00)	0.985	0.776	0.997
temporalpole surface with global thickness weight	0.635	-0.00 (-0.00, 0.00)	0.007*	0.125	0.029*
transversetemporal surface with global thickness weight	0.893	-0.00 (-0.00, 0.00)	0.462	0.634	0.574

**Figure 2:**
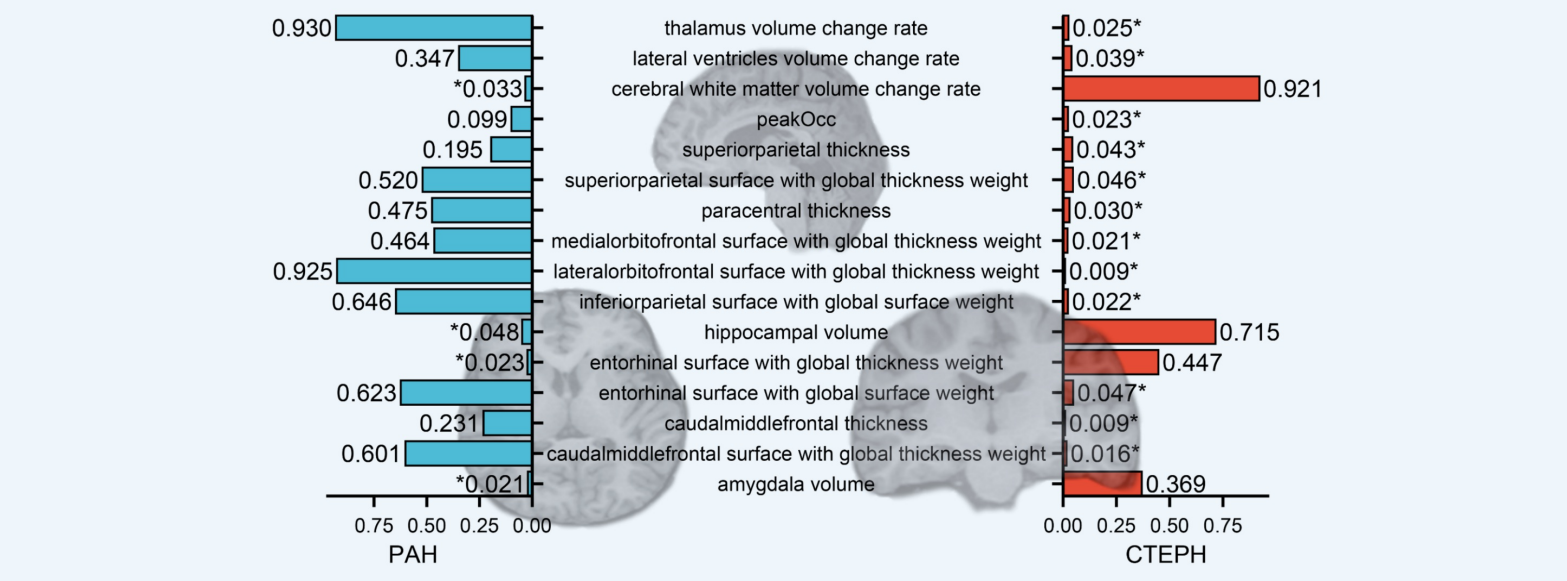
IVW-estimating p from CTEPH and PAH on brain structure on brain magnetic resonance imaging and electroencephalogram. *: p<0.05. IVW, inverse-variance weighted; CTEPH, chronic thromboembolic pulmonary hypertension; PAH, pulmonary arterial hypertension; peakOcc: occipital alpha peak frequency (associated with cognition or depression).

At the functional region-level analysis, we found a series of structural alterations on brain MRI linked to CTEPH, including the entorhinal surface (β: 1.13 mm², IVW p: 0.047) and the inferior parietal surface (β: 10.98 mm², IVW p: 0.022) with global weight (Figure 2, Tables 2, 4). It appears that the inferior parietal surface with global surface weight had resulted from CTEPH (β: 10.98 mm², IVW p: 0.022) but not PAH (β: -1.10 mm², IVW p: 0.646). Compared with PAH (β: -0.16 mm², IVW p: 0.623), the effects of CTEPH (β: 1.13 mm², IVW p: 0.047) on the entorhinal surface were relatively specific (Figure 2, Tables 2-4). For PAH, the entorhinal thickness with global thickness weight (β: -0.003, IVW p: 0.023) and hippocampal volume (β: -0.005, IVW p: 0.048) were altered specifically (Table 3).

**Table 4:** β estimates from CTEPH and PAH on genetical brain structures by Mendelian randomization using IVW *P<0.05. IVW, inverse-variance weighted; CTEPH, chronic thromboembolic pulmonary hypertension; PAH, pulmonary arterial hypertension; peakOcc: ​occipital alpha peak frequency.

Brain Structures	CTEPH, β (95% Confidence Interval)	P-value	PAH, β (95% Confidence Interval)	P-value
cerebral white matter volume change rate, mm^3^/year	-2.91 (-60.38, 54.55)	0.921	-52.72 (-101.21, -4.24)	0.033*
lateral ventricles volume change rate, mm^3^/year	22.56 (1.15, 43.97)	0.039*	-7.70 (-23.75, 8.36)	0.347
thalamus volume change rate, mm^3^/year	-7.64 (-14.31, -0.97)	0.025*	0.22 (-4.61, 5.04)	0.930
entorhinal surface, mm^2^/year	1.13 (0.01, 2.25)	0.047*	-0.16 (-0.79, 0.47)	0.623
inferiorparietal surface, mm^2^/year	10.98 (1.59, 20.37)	0.022*	-1.10 (-5.81, 3.60)	0.646
peakOcc, Hz	0.04 (0.01, 0.08)	0.023*	0.03 (-0.01, 0.06)	0.099

Furthermore, CTEPH altered the thalamus (β: -7.64 mm³/year, IVW p: 0.025) or lateral ventricle (β: 22.56 mm³/year, IVW p: 0.039) volume changing rate with aging (Figure 2, Table 1). However, there was no significant evidence of the effects of PAH (β: 0.22 mm³/year, IVW p: 0.930) on the thalamus volume aging rate or lateral ventricles volume aging rate (β: -7.70 mm³/year, IVW p: 0.347). Moreover, we also found that CTEPH was associated with the electrical activity in EEG (β: 0.04 Hz, IVW p: 0.023), but PAH had no similar association (β: 0.03 Hz, IVW p: 0.099). PAH can affect the cerebral white matter volume change rate (β: -52.72 mm³/year, IVW p: 0.033), which is different from that in patients with CTEPH.

According to Cochran's Q test, we did not find significant heterogeneity in the association between CTEPH or PAH and brain structures or electric activity mentioned above (Tables 2, 3). The MR-Egger intercept test and global test P-value derived from MR-PRESSO confirmed no horizontal pleiotropy in the causal effects of CTEPH or PAH and those brain structures or electric activity (Tables 2, 3).

Mediators of the different impacts of CTEPH and PAH on the brain structure

**Figure 3:**
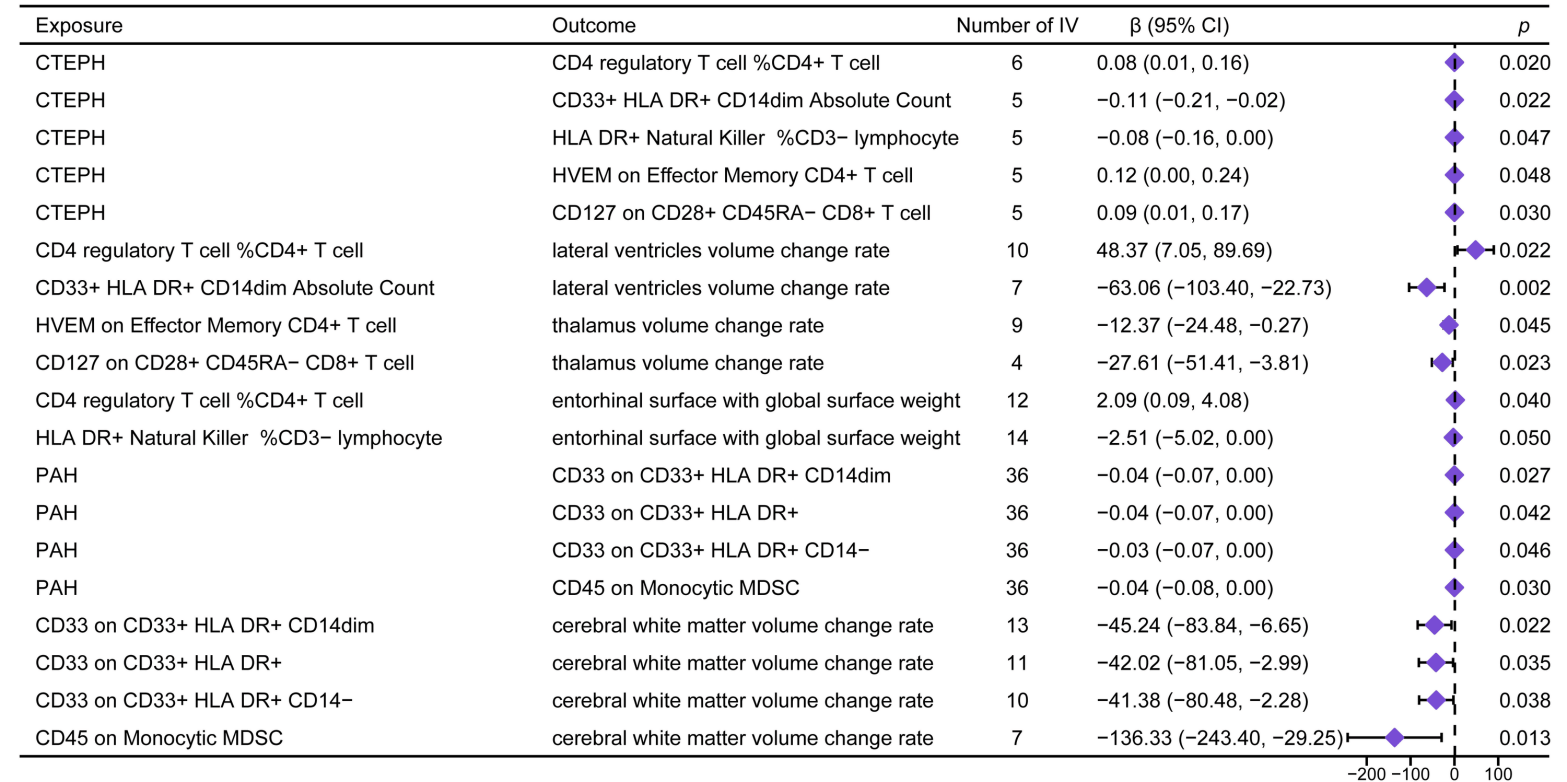
A Forest plot of potential mediators of the association between CTEPH or PAH and brain structures. β and p were derived from the inverse-variance weighted method. CTEPH, chronic thromboembolic pulmonary hypertension; PAH, pulmonary arterial hypertension; IV, instrumental variable; CI, confidence interval; MDSC, myeloid-derived suppressor cells.

Mediation pathways are graphically represented in Figure 3. CTEPH affected the absolute count of CD33+ HLA DR+ CD14dim (β: -0.11, IVW p: 0.022) and the cell count was significantly correlated with the volume change rate of the lateral ventricles (β: -63.06, IVW p: 0.002) with the mediation effect of 4.09 (confidence interval: 0.09 to 10.16). Conversely, PAH displayed a negative association with the count of CD33+ HLA DR+ CD14dim immune cells (β: -0.04, IVW p: 0.027) that was related to cerebral white matter volume change rate (β: -45.24, IVW p: 0.022) with a significant mediation effect (1.74, confidence interval: 0.01 to 4.39). Although not all mediation effects were significant in Figure 3, we observed the percentage of CD4 regulatory T cells in CD4+ T cells, the percentage of HLA DR+ natural killer cells in CD3-lymphocytes, the amount of HVEM on effector memory CD4+ T cells, and the amount of CD127 on CD28+ CD45RA-CD8+ T cells were causally changed by CTEPH, and those immune cells altered the brain structures related to CTEPH. Similarly, the amount of CD33 on CD33+ HLA DR+ cells, the amount of CD33 on CD33+ HLA DR+ CD14- cells, and the amount of CD45 on monocytic myeloid-derived suppressor cells were potential mediators of the association between PAH and the cerebral white matter volume change rate.

## Discussion

This is the first to reveal the causal effects of CTEPH and PAH on brain structures with Mendelian randomization and brain MRI. We found that both PAH and CTEPH affected brain structure and brain aging rate in two different patterns. Furthermore, our results demonstrate that circulating immune cells may mediate the association between CTEPH, PAH, and brain structures, and that immune cell activation in CTEPH patients is strongly associated with changes in brain structure distinct from those in PAH.

Earlier studies reported that PAH was associated with the inferior parietal, insula, lateral occipital, parsopercularis, parstriangularis, rostral middle frontal, and superior parietal regions [11]. Our results revealed several heterogeneous associations, likely due to data updates in the FinnGen project. The previous study used 125 PAH cases and 162837 healthy controls. In contrast, our study employed 277 cases and 316,345 controls. Although the genome-wide significance threshold in the previous study was identical to ours, the final genetic instrument variables differed substantially. Our study indicated that PAH affects hippocampal volume and amygdala volume with relatively small effect sizes. The hippocampus has been related to memory and cognition in aging diseases [19]. Since it contains high levels of glucocorticoid receptors, the hippocampus is more vulnerable to long-term stress than most other brain areas and is highly involved in severe depression [20,21]. The amygdala is activated under the conditions of fear or aggression, and it has also been linked to depression [22]. Keener et al. reported that individuals with bipolar disorder had greater amygdala activity [23]. Besides, we found that PAH decreases the rate of change in cerebral white matter volume with aging, a finding that may be similar to cognitive decline in Sturge-Weber syndrome [24]. These previous clues inspire us to conclude that our findings are evidence supporting the lung-brain axis and causal effects between PAH and affective disorders.

Previous studies [25-27] reported that the entorhinal cortex was associated with Alzheimer's disease (AD) and stable mild cognitive impairment, and the medial thalamus was associated with cognitive impairment. Our study showed that CTEPH changes the entorhinal and inferior parietal surfaces on brain T1WI MRI. CTEPH also altered the rate of change in thalamic volume with aging. Moreover, some studies have indicated that thalamic and lateral ventricular volumes naturally change with age [28], but CTEPH alters the rate of this change. Therefore, our study suggests that CTEPH promotes brain structural aging, but the details of the underlying interaction remain unclear.

PAH and CTEPH lead to right heart failure, but our data show that their impacts on the brain are different. To further study the possible mechanisms, we analyzed circulating immune cells in the effects of CTEPH and PAH on brain structural changes. T-cell activation was highlighted in the association of CTEPH and brain structures. The role of T cells in CTEPH has been highlighted in single-cell RNA sequencing and a series of human studies [29]. However, it remains unclear whether T cells can promote brain restructuring. Monocytes were implicated in the association of PAH and brain structures. Recently, monocytes have been reported to activate within pulmonary arterial subendothelial and adventitial regions, influencing vascular remodeling across PAH subtypes [30]. Our findings provide novel insights into how the lung-brain crosstalk works, inspiring future studies to validate and apply them to clinical interventions.

Combining Mendelian randomization with a brain MRI dataset is novel because it integrates genetic evidence with detailed brain structural data. This approach allows us to infer causal relationships between PH subtypes and specific brain structural changes, overcoming confounding biases present in traditional observational studies. It provides a unique opportunity to visualize and quantify how genetic predispositions to PH manifest in brain structure, offering deeper insights into the lung-brain axis. But there are limitations to this study, including the fact that the study population included only individuals of European ancestry, which makes it difficult to apply the results to other ancestral origins. As there is only one GWAS in CTEPH, future meta-analyses would be helpful to further refine the conclusions. Moreover, we studied only the effects of CTEPH and PAH on brain structure, evaluated on T1WI, and there is a lack of analysis of their effects on brain function and metabolism. Future studies may need to conduct multivariable Mendelian randomization to estimate the effects of PH on brain function using functional MRI and on metabolism using positron emission tomography.

## Conclusions

Both CTEPH and PAH can causally affect specific brain regions and electrical activity, but their impacts on functional brain regions and electrical activity differ due to their distinct etiologies and immune activations. These findings help us to understand the heterogeneity of the lung-brain axis in different subtypes of PH.
